# Exploration of antibiotic resistance risks in a veterinary teaching hospital with Oxford Nanopore long read sequencing

**DOI:** 10.1371/journal.pone.0217600

**Published:** 2019-05-30

**Authors:** Kanishka Indiwari Kamathewatta, Rhys Nathan Bushell, Neil David Young, Mark Anthony Stevenson, Helen Billman-Jacobe, Glenn Francis Browning, Marc Serge Marenda

**Affiliations:** 1 Department of Veterinary Biosciences, Faculty of Veterinary and Agricultural Sciences, The University of Melbourne, Werribee, Victoria, Australia; 2 Asia-Pacific Centre for Animal Health, Department of Veterinary Biosciences, Faculty of Veterinary and Agricultural Sciences, The University of Melbourne, Parkville, Victoria, Australia; Nankai University, CHINA

## Abstract

The Oxford Nanopore MinION DNA sequencing device can produce large amounts of long sequences, typically several kilobases, within a few hours. This long read capacity was exploited to detect antimicrobial resistance genes (ARGs) in a large veterinary teaching hospital environment, and to assess their taxonomic origin, genetic organisation and association with mobilisation markers concurrently. Samples were collected on eight occasions between November 2016 and May 2017 (inclusive) in a longitudinal study. Nanopore sequencing was performed on total DNA extracted from the samples after a minimal enrichment step in broth. Many ARGs present in the veterinary hospital environment could potentially confer resistance to antimicrobials widely used in treating infections of companion animals, including aminoglycosides, extended-spectrum beta-lactams, sulphonamides, macrolides, and tetracyclines. High-risk ARGs, defined here as single or multiple ARGs associated with pathogenic bacterial species or with mobile genetic elements, were shared between the intensive care unit (ICU) patient cages, a dedicated laundry trolley and a floor cleaning mop-bucket. By contrast, a floor surface from an office corridor without animal contact and located outside the veterinary hospital did not contain such high-risk ARGs. Relative abundances of high-risk ARGs and co-localisation of these genes on the same sequence read were higher in the laundry trolley and mop bucket samples, compared to the ICU cages, suggesting that amplification of ARGs is likely to occur in the collection points for hospital waste. These findings have prompted the implementation of targeted intervention measures in the veterinary hospital to mitigate the risks of transferring clinically important ARGs between sites and to improve biosecurity practices in the facility.

## Introduction

In bacterial populations, the resistome is defined as “the collection of all genes that could contribute to a phenotype of antibiotic resistance” [1]. In human hospitals, monitoring of patient and environmental microbiomes has revealed complex patterns of surface colonisation and pervasive exchanges of resistance genes [2, 3]. While animal-associated microbiomes and resistomes have been studied in livestock flora [4, 5] as well as in farm environments and effluents [6–9], relatively few investigations have been carried out in veterinary hospitals. Large veterinary teaching hospitals accommodate a transient population of animals with various resident flora, infectious status, and previous exposure histories to antibiotics. This unique environment could act as a mixing reservoir for antimicrobial resistance genes (ARGs) to and from various sources including humans and animals. Most veterinary teaching hospitals have active and passive surveillance programs to monitor infectious risks and antimicrobial resistance trends associated with their patients [10], but the presence and diversity of ARGs in the hospital environment is often not explored. Therefore, we sought to determine the presence of ARGs in such environmental systems and to map their associations with their bacterial hosts or mobile genetic elements (MGEs).

Surveillance studies for ARGs have benefited from the rapid advances in next generation sequencing (NGS) technologies, which overcome the composition bias or limited detection capacity commonly associated with culture or PCR based methods [11, 12]. The Illumina NGS technology is cost effective and has low error rates, but its shorter read length makes the assembly and analysis of contiguous genomic sequence regions containing several, co-localised ARGs and/or MGEs challenging [13]. In contrast, long reads produced by the Oxford Nanopore Technologies MinION portable sequencer [14] readily permit the discovery of co-localised ARGs and their flanking genomic nucleotide sequences. This additional information is critical for correctly assigning reads to their taxonomic classification node and to provide insights into the bacterial host of the ARGs that are detected. The MinION sequencing technology has been employed previously to profile the gut resistome of a human patient, by functional metagenomics analysis of a plasmid expression library [15]. However, nanopore sequencing has not been applied to resistome analysis directly in veterinary environmental samples.

Here, a reproducible protocol was established to explore the resistomes associated with a veterinary hospital environment and to attribute the detected ARGs to their corresponding bacterial hosts or genetic origin using nanopore sequencing. The taxonomic classifications and quantitative detection of ARGs from MinION data were compared to reference methods, i.e. 16S rRNA analysis, WaferGen qPCR arrays for ARGs, Illumina sequencing and bacteriological culture surveys, to confirm the nanopore sequencing results. Finally, four MinION long read datasets obtained from different parts of the veterinary hospital environment were analysed to evaluate the significance of ARG profiles in relation to their clinical risk, co-occurrence in their bacterial host and potential for transmission between microorganisms, with the aim of improving infection control procedures in veterinary facilities.

## Materials and methods

### Sampling

This longitudinal study was carried out in the U-Vet Werribee Animal Hospital of The University of Melbourne between November 2016 and May 2017 (inclusive). Sampling was conducted across four environmental sources. The inner surfaces of patient cages in the intensive care unit (ICU cages) and the inner surfaces of a plastic laundry trolley (LT) were sampled on eight independent occasions. The trolley, which is used to collect soiled bedding from the ICU cages but is kept outside the ICU room, was swabbed on the same days as the ICU cages. The liquid contents in a mop bucket (MB) used to clean the ICU floor were collected on eleven independent occasions. The floor of an office corridor (OC) with controlled access, no animal contact and limited foot traffic by the veterinary hospital staff was sampled once. A sterile gauze swab was moistened with buffered peptone water (BPW, Oxoid) and used to wipe approximately 1 m^2^ from the hard surfaces (ICU cages, LT, OC). On each occasion an average of 4 (range 2 to 5) occupied ICU cages were swabbed and the swabs were pooled in a single sterile container. Pooled ICU cage swabs and single swabs from the LT and OC were transferred to the laboratory within 5 to 10 minutes of collection and processed immediately. The swabs were mixed with 100 mL BPW. The liquid contents (50 mL) from the MB were collected using sterile syringes, transferred into sterile containers and 75 ml of BPW was added. Aliquots of the suspensions were removed for culture (see below) and 20 mL aliquots were incubated at 30°C for 16 hours without shaking to reach an OD_600nm_ of 0.8.

### Bacterial cultures and phenotypic testing for antibiotic resistance

Aliquots (100 μL) from sample suspensions were ten-fold diluted with BPW and plated on Mueller Hinton (MH) agar without antibiotics, to estimate the total counts of viable bacteria. Undiluted sample suspensions (100 μL) were plated on MH agar containing ampicillin 50 μg/mL, enrofloxacin 2 μg/mL and gentamicin 15 μg/mL to select for multi-drug resistant organisms (MDR), and on Brilliance ESBL agar (Oxoid, UK) to select for extended spectrum beta lactamase (ESBL) producing organisms. The antibiotic concentrations used in MH agar plates were chosen empirically, based on routine antimicrobial susceptibility testing results observed in our diagnostic laboratory and the EUCAST database of MIC distributions in common pathogenic bacteria, available from the organisation website (http://www.eucast.org/). The plates were incubated for 24 to 48 hours at 37°C.

### DNA extraction

Cells from 20 mL of BPW cultures were collected by centrifugation at 1700 × *g* for 30 minutes at 4°C on Allegra X-22R centrifuge with SX4250 swing bucket rotor (Beckman Coulter, USA). Genomic DNA was extracted from the cell pellets with the Wizard Genomic DNA Purification Kit (Promega, Madison, WI). After resuspension, one half of the cells were processed with the manufacturer’s recommended protocol for Gram positive bacteria while the other half were processed with the protocol for Gram negative bacteria. The DNA concentration was measured by Qubit fluorometer (Invitrogen, USA) and the quality was determined by microspectrophotometry (NanoDrop ND-1000, NanoDrop technologies, Wilmington, DE). For each sampling occasion, equal volumes of Gram positive and Gram negative bacterial DNA extracts were mixed and cleaned by 1× SPRI beads (AMPureX, Beckman Coulter, CA, USA).

### Library preparation, sequencing and read processing

The MinION sequencing libraries were prepared with the 1D genomic DNA sequencing kit SQK-LSK108 (Oxford Nanopore Technologies (ONT), UK). Briefly, 1–4 μg DNA was sheared into 8 kb fragments using g-tubes (Covaris, Brighton, UK) at 2539 × *g* for 1 minute, nicks were repaired with the Formalin-Fixed Paraffin-Embedded (FFPE) enzyme mix (New England BioLabs, USA), followed by end repair, dA-tailing and adaptor ligation, with or without barcoding. The sequencing was done in a portable MinION sequencing device, with R9.4 or R9.5 flow cells (ONT, UK). Raw reads were basecalled into fastq or fast5 files with the program Albacore version 2.1.7 (ONT, UK) unless specified otherwise. The fast5 files were converted to fastq format using poretools version 0.6.0 [16]. Sequences were filtered with the program Fast5-to-Fastq [17] to select reads with a Phred quality score of ≥8 and read length of ≥250 bases. Filtered reads were then converted into fasta format using the program Fastaq [18]. A detailed explanation of the sequencing runs schedule is given in the S2 Table.

The Illumina sequencing libraries were prepared according to the TruSeq DNA v1.0 protocol. First, the DNA ends were repaired followed by A-tailing, index and adaptor ligation. Then the ligated libraries were enriched by PCR. Finally, the indexed and enriched libraries were pooled before sequencing in a NextSeq benchtop sequencer to obtain paired-end reads of 150 bp (Walter and Eliza Hall Institute of Medical Research, Victoria, Australia). The program Trim Galore version 0.4.4 [19] was used to remove the adaptors from the reads and to filter out the reads having a Phred quality score of <20. The adaptor trimmed and quality filtered fastq reads were assembled using the MEGAHIT version 1.1.4 [20].

### Evaluation of microbial community composition

The Kraken taxonomic classifier version 1 [21] was used with the MiniKraken 2014 database [22] which contained complete bacterial, archaeal and viral genomes from RefSeq. Kraken outputs were then converted to show full taxonomic lineages using the script kraken-translate [21]. The program Centrifuge [23] was used with its own indexed bacterial and archaeal database. After taxonomic classification, reads with Centrifuge quality scores of less than 300 and less than 50 bp match lengths [23] were filtered out. Full taxonomic lineage for the Centrifuge output (NCBI taxonomic IDs) were obtained from the NCBI taxonomy website [24] and reports were combined using R version 3.4.0 [25]. Rarefaction curves were computed using the Kraken and Centrifuge outputs and using the contributed ‘vegan’ package version 2.4–5 [26] in R.

The 16S rRNA sequencing and diversity profiling was performed by the Australian Genome Research Facility (AGRF, Australia). The V3 and V4 hypervariable regions of 16S rRNA gene were PCR amplified using established universal forward 341F (CCTAYGGGRBGCASCAG) and reverse 806R (GGACTACNNGGGTATCTAAT) primers [27, 28]. The V1 and V3 hypervariable regions were amplified using established universal forward 27F (AGAGTTTGATCMTGGCTCAG) and reverse 519R (GWATTACCGCGGCKGCTG) primers [29]. The Illumina MiSeq platform was used with Illumina’s Nextera XT Index Kit and Paired End Sequencing Chemistry. The sequence reads were analysed as described previously [30]. Briefly, overlapping paired reads were merged by aligning forward and reverse reads using USEARCH 8.1 [31] and seqtk toolkit was used to trim the primer sequences from the read-ends [32]. Then merged reads were filtered for length >400 bp and quality of <1 expected error [33], followed by clustering into operational taxonomic units (OTUs) [34] using USEARCH 8.1 [31]. The QIIME 1.9.1 pipeline was used to assign the representative sequences from each OTU into relevant taxa [35]. The taxonomy assignment script “assign_taxonomy.py” from QIIME was used with the Greengenes database [36] version 13_5.

### Detection and quantification of antimicrobial resistance genes (ARGs)

ARGs were identified within MinION long read fasta sequences and Illumina contigs using ABRicate [37] and the following databases: (i) in-built antimicrobial resistance gene database, Resfinder [38], which contain 2228 ARG sequences; (ii) simplified/non-redundant subset of Resfinder ARGs containing 646 sequences; and (iii) a custom database of 16S rRNA sequences used to normalise the ARG counts. The non-redundant Resfinder ARG subset was constructed by clustering the Resfinder ARG database sequences with >90% nucleotide identity using the package CD-HIT-EST in program CD-HIT [39]. The custom-made 16S rRNA database (rdpBac) was derived from 11,988 16S rRNA gene sequences downloaded from the Ribosomal Database Project [40], release 11. The BLAST hits having <70% coverage and <80% identity with the gene sequences from ABRicate databases were excluded from the analysis.

DNA samples were further analysed with a high capacity WaferGen SmartChip Real-time qPCR array (WaferGen Biosystems, Fremont, CA, USA). In this array, a total of 296 validated primer sets were used, including 285 primers targeting ARGs, 10 primers targeting MGEs and 1 primer targeting 16S rRNA gene [41, 42]. The relative abundances of ARGs and MGEs were calculated relative to the 16S rRNA gene using the 2^-ΔCT^ method [43].

### Taxonomic classification of individual long reads containing ARGs

For each sample, the standard kraken-translate output file, which contains the sequence read ID and the assigned taxonomic lineage was merged with each ABRicate ARG output file using the sequence read ID as the key in R version 3.4.0 [25]. The combined output was used to attribute ARGs to their corresponding bacterial hosts that were detected in the veterinary hospital environmental samples. Bubble plots were constructed with the package ggplot2 version 3.1.0 in RStudio version 1.0.143. Reads carrying ARGs known to occur in MGEs and reads carrying multiple ARGs were analysed using the BLASTN function in ISfinder server using a cut-off e-value of 1e^-5^ [44]. Nucleotide BLAST version 2.2.31+ was used to search a local BLAST nucleotide database of 8675 sequences, made by combining all antibiotic resistance genes, virulence factors and plasmids sequences provided by the program ABRicate [37]. The software Artemis [45] was used to annotate the reads based on the ISfinder and BLASTN outputs.

### Statistical comparison of WaferGen and MinION results

The primersearch function in Jemboss 1.5 [46] was used to search WaferGen primer pairs with matches in the DNA sequences in Resfinder database. For each ARG having a representative sequence in ResFinder and a cognate PCR primer pair in the WaferGen array, the gene abundance was first normalised against the 16S rRNA gene abundance values calculated from the long reads analysis and the WaferGen results. Then, the differences between each of the normalised gene abundances and the 16S rRNA gene abundance were compared using Spearman’s rank correlation coefficient in R version 3.4.0.

## Results and discussion

### Larger amounts of input DNA result in higher sequencing yields

The surface swabs collected throughout the veterinary hospital typically contained 10^4^ to 10^5^ total bacteria, based on colony counts on MH plates without antibiotics. As these samples did not provide sufficient biomass to extract the recommended amount of DNA for a standard MinION library preparation (at least 1 μg), the bacterial population was amplified by an incubation step for 16 hours at 30°C in BPW. While this step is expected to reduce the taxonomic diversity of the initial sample, it was deemed that bacteria usually associated with major infectious risks in veterinary hospitals (e.g. *Enterobacteriaceae*, *Staphylococcus* sp.) are also the most likely to be recovered by this enrichment process and are therefore the most relevant organisms to consider in this study.

Different strategies for library preparation were tested to optimise the yield of sequenced long reads per run, using DNA extracts from the ICU cages and LT in 3 and 2 independent sequencing reactions, respectively. Increasing the amount of DNA in the pre-sequencing mix well above the recommended minimum of ~50–200 ng (up to ~900 ng) resulted in a proportional expansion of the total sequence outputs in Gbp over a comparable time frame of 16 hours (Table 1). Thus, high DNA input, along with the advantages of nanopore sequencing libraries such as reduced preparation time and the longer read lengths [47] should allow to capture as many as possible microbial genomic data from the sample, a desirable aim in this type of study [48].

**Table 1:** Input DNA amounts versus sequencing yields.

Sequencing library	DNA amount used for preparing library (μg)	DNA amount loaded on flow cell (ng)	Flow cell chemistry	Run time (hours)	Cumulative yield (gigabases)^a^	Cumulative yield (number of reads)^a^
ICU1	0.7	174	R 9.4	16	0.87	175,000
ICU2	1.2	322	R 9.5	27	1.45	275,000
ICU3	4	630	R 9.5	48	2.75	600,000
LT1	1.2	316	R 9.4	16	1.20	175,000
LT2	4.9	910	R 9.5	16	4.15	750,000

^a^ values recorded after 16 hours of sequencing

### Nanopore sequencing is a reliable method to explore environmental microbiota

To validate the taxonomic assignments obtained with MinION sequencing, the microbial compositions of two aggregates of 19 ICU cage swabs and 4 LT swabs, were inferred from MinION long read data using the Kraken or Centrifuge classifiers and compared to sequence analysis results of 16S rRNA regions V1-V3 and V3-V4 from the same DNA extracts.

In the ICU cages and LT, all four methods (MinION-Kraken, MinION-Centrifuge, 16S/V1-V3, 16S/V3-V4) detected a similar composition of taxa at the phylum, class, and order levels (Table 2). At the family level, the four methods were concordant except for reads assigned to the family *Enterococcaceae* in the ICU cages, which composed approximately 21% of the reads according the MinION-Kraken, MinION-Centrifuge and 16S/V3-V4 but only 2% with the 16S/V1-V3 analysis. At the genus level, the MinION-Kraken and MinION-Centrifuge assigned taxonomic classification to 83% and 94% of the ICU cages reads, respectively. In contrast, 16S/V1-V3 and 16S/V3-V4 sequencing of the ICU cages sample successfully classified only 22% and 37% of the reads, respectively; a similar pattern was observed for the LT samples (Table 2). These results confirmed previous studies suggesting that long read nanopore sequencing provides better resolution at lower taxonomic levels compared to amplicon-based, short-read 16S rRNA sequencing [49–52]. Moreover, there was a lower agreement between 16S/V3-V4 and 16S/V1-V3 rRNA sequencing than between long read classifier tools, possibly due to known amplification biases between the two conserved 16S rRNA primer sets [53]. These results were confirmed by comparing the MinION long reads directly obtained from the ICU cages and LT against the MEGAHIT contigs assembled from Illumina reads using the same DNA preparations. The Kraken taxonomic assignments for both datasets were in agreement, further demonstrating the reliability of the MinION-Kraken approach.

**Table 2:** Percentages of reads assigned at different taxonomic levels with four classification methods.

Taxa	% of reads assigned a taxonomic classification^a^							
	ICU cages				Laundry trolley			
	16S/ V1-V3	16S/ V3-V4	MinION/ Kraken	MinION/ Centrifuge	16S/ V1-V3	16S/ V3-V4	MinION/ Kraken	MinION/ Centrifuge
**Phylum**								
*Proteobacteria*	69	53	60	62	73	60	71	70
*Firmicutes*	31	47	40	38	27	40	29	30
**Class**								
*Bacilli*	27	42	39	37	18	30	26	27
*Clostridia*	5	5	1	1	9	10	3	3
*Gammaproteobacteria*	69	53	59	62	72	60	70	70
**Order**								
*Bacillales*	13	22	17	17	17	29	23	25
*Clostridiales*	5	5	1	1	9	10	3	3
*Enterobacteriales*	57	44	50	51	71	59	68	68
*Lactobacillales*	14	20	22	20	1	2	3	2
*Pseudomonadales*	11	9	9	10	1	1	2	2
**Family**								
*Bacillaceae*	9	16	14	14	14	22	22	24
*Clostridiaceae*	3	3	1	1	4	5	3	3
*Enterobacteriaceae*	57	44	50	47	71	59	68	67
*Enterococcaceae*	2	20	22	20	1	2	3	2
*Peptostreptococcaceae*	2	2	0	0	5	4	0	0
*Planococcaceae*	2	5	0	0	3	6	0	0
*Pseudomonadaceae*	11	9	9	10	1	1	1	2
*Staphylococcaceae*	1	1	3	3	0	0	1	1
Unassigned	12	0	1	0	0	0	1	0
**Genus**								
Assigned	22	37	83	94	10	39	92	99
Unassigned	78	63	17	6	90	61	8	1

^a^ data for <1% assignments with all four methods in both samples are not shown in the table

A prior knowledge of the microbial community composition can improve 16S rRNA experimental design [54] but this information is not always available. Moreover, in a veterinary hospital environment, microbial communities are expected to represent several interconnected microbial eco-systems. As an example, the patient cages in ICU may contain microorganisms coming from animal skin, saliva, urine and faeces together with soil and other environmental microorganisms. These organisms contaminate bedding which in turn can contaminate the laundry trolley, and the mops used to clean the floor of the room. This makes nanopore sequencing an attractive approach to track ARGs and infectious risks in veterinary hospitals.

### Detection and quantification of ARGs by MinION long read sequencing is confirmed by targeted qPCR assays

To assess the capacity of long read data to accurately detect the most abundant ARGs within complex environments, the DNA extracts from an aggregate of 19 ICU cages and 4 LT swabs (see S2 Table for details) were analysed with MinION and WaferGen technologies. The MinION sequence reads were searched for ARGs by the program ABRicate with the database Resfinder containing 2228 sequences. Read statistics for the MinION datasets are reported in S3 Table. Separately, the same DNAs were amplified in a WaferGen qPCR array with 296 gene-specific primer pairs. Only the sequence targets represented in both methods were compared.

**Fig 1:**
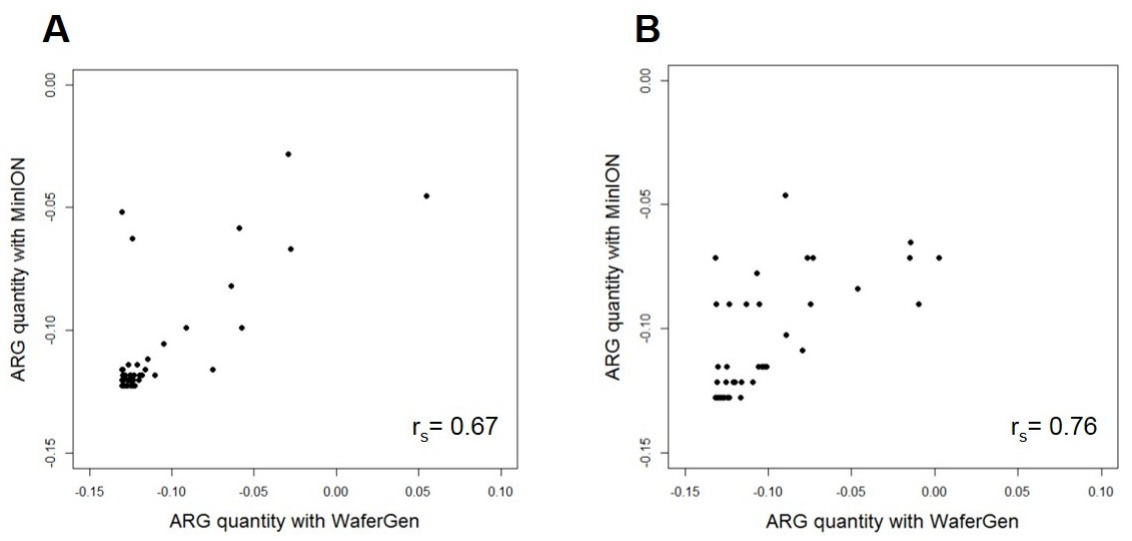
Comparison of WaferGen ARG 2^-ΔCT^ values with MinION ARG counts (hits) with reference to the 16S rRNA gene. (A) ICU cages (B) Laundry Trolley. X axis: normalised differences in 2^-ΔCT^ values of ARGs when compared to 16S rRNA 2^-ΔCT^ value obtained with WaferGen; Y axis: normalised differences in counts of ARGs when compared to the 16S rRNA count obtained with program ABRicate using MinION sequence data.

Respectively, 93% and 97% of the ARGs detected in ICU cages and LT samples by MinION sequencing were also found with the WaferGen qPCR array. The normalised abundances of ARGs relative to the 16S rRNA gene were highly correlated between the two methods, with a Spearman’s rank correlation coefficient of 0.67 for the ICU cages and 0.76 for the LT (Fig 1). The WaferGen qPCR array detected respectively 66 and 60 ARGs with a Ct value of 27 in the ICU cages and LT samples. Out of those, respectively 39 and 28 genes were also found in the MinION sequence datasets. The ARGs that were not found with the MinION sequencing but were detected by the WaferGen qPCR array were present in the sample at a very low relative abundance (0.08% or below) in the population, suggesting that qPCR methods were more sensitive for detecting low abundance ARG targets.

However, respectively 37/107 (35%) and 26/59 (44%) ARGs detected in the ICU cages and LT by MinION sequencing were not included in the WaferGen array. Some of these genes, such as *blaOXA*, confer resistance to clinically important beta lactam antibiotics and are known to be present on MGE as well [55]. Although different primer pairs could be included, the array capacity is currently limited to a maximum of 384 reactions, underlining the advantage of using a metagenomic approach to detect ARGs in complex samples. While nanopore sequencing has a low accuracy (typically 80–90%), it is expected that the relatively random distribution of errors within reads should not adversely impact the identification of most ARGs by ABRicate, which is based on BLASTN alignments and can tolerate some degree of sequence mismatches. This hypothesis was supported by examining alignments between individual nanopore reads that putatively contained ARGs and corresponding sequences from the resfinder database. Although a number of mismatches, attributable to sequencing errors, affected the overlapping region of the published ARG and the long read fragment, the alignement scores clearly indicated the presence of an ARG in the read.

### A majority of environmental ARGs can be linked to a bacterial genus or a mobile genetic element

A recently developed ranking scheme [56] has proposed that ARGs found in a clinically relevant pathogen or in a MGE should be classified as high-risk ARGs, while resistances known to be intrinsic for a particular bacterial species will be considered only as markers for the presence of those bacteria. All reads containing ARGs were searched for information on their taxonomic or genetic origin in the adjacent sequences to evaluate the risks associated with ARGs in the veterinary hospital environment. By using this approach, it is possible to detect the bacterial host of the ARGs present on chromosomal sequences which are long enough to assign a taxonomic origin to the read. Although this method does not warrant the precise taxonomic identification of the bacterial hosts of plasmid sequences, it could point to a group of prokaryotes as the presumptive host for the element, and its potential for dissemination in bacterial populations.

**Fig 2:**
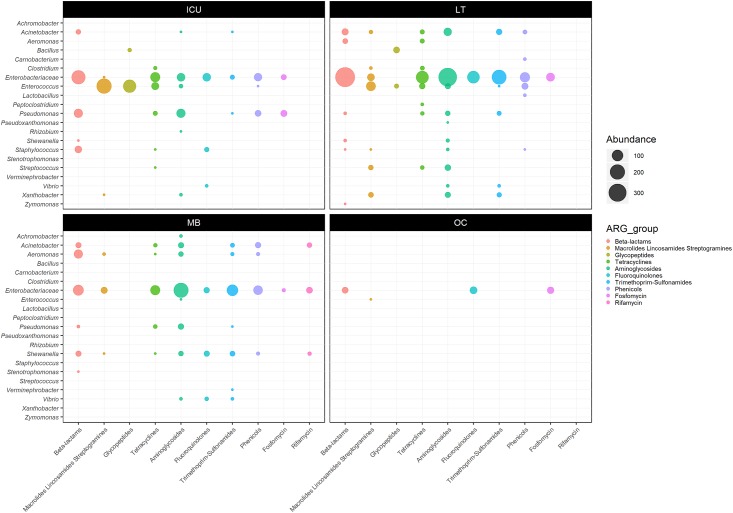
Abundance of bacterial reads carrying ARGs relative to their taxonomic origin in the ICU cages (ICU), Laundry Trolley (LT), Mop Bucket (MB) and Office Corridor (OC).

Overall, 77% (1001/1299), 65% (1688/2592), 38% (813/2166) and 99% (80/81) of the ARGs detected respectively in the ICU cages, LT, MB and OC samples were also classified into a bacterial genus or family by the program Kraken. The lower rate of taxonomic assignment in MB sample might be due to the shorter read lengths observed in that particular sequencing library (i.e. mean read length of ~2 kb as opposed to ~5 kb in the other libraries). Many ARG-carrying taxons identified in the veterinary hospital fell into groups commonly found in animal flora, e.g. *Enterobacteriaceae*, *Enterococccus* and *Staphylococcus* (Fig 2 and S1 Table). The taxonomic classifications assigned by the Kraken and Centrifuge at the genus level were mostly consistent, except for some ARG-containing reads that were assigned by the two programs to different genera of *Enterobacteriaceae*. To ensure accuracy, these reads were reported at the family level only.

Respectively 23%, 67% and 65% of the ARGs-containing reads from the ICU cages, LT and MB samples also carried nucleotide sequences matching transposons, transposases and/or insertion sequences. Furthermore, the majority of aminoglycoside, tetracycline, trimethoprim or sulphonamide resistance genes were associated to MGEs (Table 3). Respectively 32%, 53% and 24% of beta lactam resistance genes detected in the ICU, LT and MB samples were classified as Extended Spectrum Beta Lactamases (ESBLs). Again, most of these ESBL genes were also located next to MGEs, with the exception of the carbapenem resistance gene *imiS*, which belongs to Ambler Class B metallo-beta-lactamases [57], found in the MB sample.

**Table 3:** Antimicrobial resistance genes associated with mobile genetic elements in the veterinary hospital.

Gene	n (N)^a^ in Sample^b^		
	ICU	LT	MB
**AACs**^c^	**4 (4)**	**76 (77)**	**49 (70)**
*aac(3)-IIa*	-	19 (20)	28 (30)
*aac(3)-IVa*	-	4 (4)	-
*aac(6')-aph(2'')*	-	14 (14)	-
*aac(6')-IIc*	4 (4)	38 (38)	1 (1)
*aac(3)-VIa*	-	1 (1)	-
*aac(6')-Ic*	-	-	20 (39)
**ANTs**^c^	**5 (5)**	**71 (71)**	**19 (19)**
*aadA1*	-	24 (24)	18 (18)
*aadA2*	2 (2)	32 (32)	1 (1)
*aadB*	-	3 (3)	-
*ant(6)-Ia*	3 (3)	12 (12)	-
**APHs**^c^	**66 (71)**	**278 (278)**	**159 (159)**
*aph(3')-Ia*	4 (4)	77 (77)	4 (4)
*aph(3')-IIa*	24 (25)	-	-
*aph(3')-III*	2 (2)	12 (12)	-
*aph(4)-Ia*	-	6 (6)	-
*aph(6)-Ic*	24 (28)	-	-
*aph(3')-VIa*	-	2 (2)	-
*strA*	6 (6)	85 (85)	136 (136)
*strB*	6 (6)	96 (96)	19 (19)
**ESBLs**^c^	**84 (84)**	**208 (221)**	**20 (42)**
*blaSHV*	1 (1)	48 (61)	-
*blaTEM*	82 (82)	157 (157)	20 (20)
*blaCARB*	-	1 (1)	-
*imiS*	-	-	0 (22)
*blaCTX-M-101*	-	2 (2)	-
*blaCTX-M-110*	1 (1)	-	-
**CATs**^c^	**3 (3)**	**80 (82)**	**63 (85)**
*cat(pC194)*	-	14 (14)	-
*cat(pC233)*	1 (1)	12 (14)	-
*catA1*	1 (1)	8 (8)	-
*catA2*	1 (1)	37 (37)	14 (34)
*catB8*	-	9 (9)	46 (47)
*catB3*	-	-	3 (4)
**DFRs**^c^	**3 (3)**	**59 (59)**	**5 (5)**
*dfrA1*	-	5 (5)	-
*dfrA12*	-	12 (12)	-
*dfrA18*	-	15 (15)	-
*dfrA30*	3 (3)	11 (11)	-
*dfrA32*	-	2 (2)	-
*dfrA7*	-	10 (10)	-
*dfrA10*	-	1 (1)	-
*dfrA13*	-	1 (1)	-
*dfrA15*	-	2 (2)	-
*dfrA14*	-	-	5 (5)
**MLSs**^c^	**3 (3)**	**62 (64)**	**14 (14)**
*ere(A)*	2 (2)	29 (29)	1 (1)
*mph(A)*	-	6 (6)	12 (12)
*mph(E)*	-	1 (2)	-
*erm(Q)*	-	1 (1)	-
*erm(B)*	1 (1)	22 (23)	-
*msr(E)*	-	2 (2)	1 (1)
*lnu(B)*	-	1 (1)	-
**QNRs**^c^	**2 (17)**	**35 (36)**	**13 (20)**
*QnrB10*	0 (5)	20 (21)	-
*QnrB12*	0 (10)	11 (11)	-
*QnrS1*	-	4 (4)	-
*QnrVC3*	2 (2)	-	13 (20)
**SULs**^c^	**6 (6)**	**162 (163)**	**109 (118)**
*sul1*	2 (2)	134 (134)	29 (29)
*sul2*	4 (4)	28 (29)	80 (89)
**TETs**^c^	**54 (68)**	**108 (124)**	**46 (48)**
*tet(A)*	21 (21)	10 (10)	17 (17)
*tet(B)*	9 (9)	49 (49)	26 (28)
*tet(C)*	-	3 (3)	1 (1)
*tet(D)*	1 (1)	29 (33)	1 (1)
*tet(E)*	-	5 (7)	1 (1)
*tet(L)*	0 (1)	7 (10)	-
*tet(M)*	0 (4)	4 (11)	-
*tet(O)*	23 (32)	-	-
*tet(X)*	-	1 (1)	-
**ARR**^c^	**-**	**-**	**33 (34)**
*ARR-3*	-	-	33 (34)
**Total**	**228 (262)**	**1139 (1175)**	**530 (614)**

^a^ Read counts: n = number of sequences showing significant similarities with the sequences of known MGEs; N = number of total sequences assigned to a particular ARG by ABRicate

^b^ Sample: ICU: Intensive Care Unit; LT: Laundry Trolley; MB: Mop Bucket. ISFinder e-value: 1×10^−5^

^c^ Presence confirmed with Illumina sequencing

AACs: aminoglycoside acetyltransferases; ANTs: aminoglycoside nucleotidyltransferases; APHs: aminoglycoside phosphotransferases; ESBLs: extended spectrum beta-lactamases; CATs: chloramphenicol acetyltransferases; DFRs: dihydrofolate reductases; MLSs: macrolides, lincosamides, and streptogramins resistance genes; QNRs: quinolone resistance genes; SULs: sulfonamide resistance genes; TETs: tetracycline resistance genes; ARR: rifampin ADP-ribosyltransferase

In addition to high-risk ARGs, intrinsic resistance genes were also detected in all four samples. For instance in the ICU cages sample, 178 sequences classified into *Enterococcus faecalis* carried the *lsa* gene [58]. Similarly, 11 sequences matching the *msrC* gene were detected in reads classified into *Enterococcus faecium* and 146 vancomycin resistance genes were carried by the *Enterococcus casseliflavus* intrinsic gene cluster *vanC*, *vanRC*, *vanSC*, *vanTC and vanXYC* [58]. Moreover, the *blaOXA-50* which occur naturally in *Pseudomonas aeruginosa* [59, 60] and *blaLEN* genes which are present on the chromosome of the *Klebsiella pneumoniae* isolates and lack the ability to develop into ESBL genes [61, 62] were identified.

Finally, none of the clinically relevant ARGs found in the veterinary hospital were detected in the OC. In this sample, 13 *oqxA* and 18 *oqxB* genes carried by *Klebsiella pneumoniae* were found, confirming that this species is a reservoir for *oqxAB* [63, 64]. The *fosA* resistance gene in *Klebsiella* and *Enterobacter* species [65] was also detected. These ARGs were located on chromosomal sequences and were not associated with MGEs. By contrast, the Illumina/MEGAHIT contigs which carried ARGs were often too short to provide the same level of insight on their taxonomic or genetic origin.

These results must take into account that using nanopore sequencing alone could underestimate the diversity of functional ARGs in the sample. Given the error rates of this method, it might not be possible to differentiate highly similar ARGs derived from a common ancestor. To address this issue, a thorough analysis of metagenomic sequence assemblies that combine long nanopore reads and short accurate Illumina reads is required.

### Phenotypic resistance patterns of bacteria recovered from the veterinary hospital environment support MinION sequencing results

Bacteriological cultures of environmental samples from the veterinary teaching hospital confirmed the presence of bacteria displaying multiple drug resistance (MDR) to ampicillin, enrofloxacin and gentamicin, as well as ESBL producing phenotypes, which were predicted by the MinION sequencing data from the same samples (Table 4). As an example, a MDR and ESBL producing *Klebsiella* species was isolated from the LT sample in which the *Klebsiella*-associated ARGs *qnrB*, *aadA* and *blaSHV* sequences were detected. In the MB sample, oxidase positive Gram negative rods with a MDR phenotype were grown on selective agar plates, in accordance with the finding of sequencing reads classified as *Pseudomonas*, *Aeromonas* and *Shewanella* species carrying enrofloxacin, gentamicin and ampicillin ARGs. Finally, *Staphylococcus* species resistant to enrofloxacin and ampicillin were detected in the ICU cages sample by culture; MinION results also indicated the presence of *Staphylococcus* species carrying *norA* and *blaZ* genes. While the complete bacteriological identification of all species cultured from these samples was beyond the scope of this study, these preliminary observations broadly support the use of nanopore sequencing data as a means for monitoring bacterial resistance profiles in a veterinary hospital environment.

**Table 4:** Comparison of phenotypic and genotypic detection of antimicrobial resistance in the veterinary hospital.

Sample	Conventional bacteriology		MinION sequencing	
	Organism	Resistance phenotype	ARG (ABRicate)	Genus (Kraken)
ICU	*Enterococcus*	MDR	*(aph(3')-III; ant(6)-Ia)*^a^	*Enterococcus*
	*Staphylococcus*	MDR	*blaZ*	*Staphylococcus*
			*norA*	
LT	*Klebsiella*	MDR ESBL	*(QnrB; aadA; blaSHV)*^a^	*Klebsiella*
			*(blaCTX-M; QnrS)*^a^	
			*(blaTEM; aac(3)-IIa)*^a^	
			*(strA; strB; aph(3’)-Ia; blaSHV; aadA)*^a^	
	*Enterobacteriaceae*	MDR ESBL	*(aadA; aac(3)-VIa; QnrB; blaTEM; blaOXA; aadA)*^a^	*Salmonella*
			*(blaTEM; strA; strB; aph(3’)-Ia)*^a^	
			*(strB; strA; QnrB)*^a^	
			*(QnrB; blaDHA)*^a^	*Escherichia*
			*(aph(3’)-Ia; strA; strB)*^a^	
			*blaTEM*	
			*(blaTEM; strB; strA)*^a^	*Citrobacter*
			*blaCMY*	
			*QnrB*	
			*(blaCMY; strA; strB)*^a^	*Enterobacter*
			*QnrB*	
	*Enterococcus*	MDR	*(aph(3’)-III; ant(6)-Ia*, *aac(6’)-aph(2”))*^a^	*Enterococcus*
MB	Oxidase positive Gram negative rods	MDR	*(strA; strB)*^a^	*Pseudomonas*
			*aac(3)-IIa*	
			*blaTEM*	
			*(aac(3)-IIa; QnrVC3)*^a^	*Shewanella*
			*(blaOXA; aadA)*^a^	
			*(blaTEM; aac(3)-IIa)*^a^	*Aeromonas*
			*imiS*	
			*(strA; strB)*^a^	
			*blaOXA*	
			*blaMOX*	
			*blaFOX*	
	KESC group	ESBL	*blaTEM*	*Klebsiella* *Enterobacter* *Serratia*

^a^ multiple ARGs on the same sequence read. MDR; multidrug resistance to Ampicillin = > 50 μg/ml: Enrofloxacin = > 2 μg/ml: Gentamicin = > 15 μg/ml. ESBL; extended spectrum beta lactam resistance. KESC; *Klebsiella*, *Enterobacter*, *Serratia*, *Citrobacter*. ICU: Intensive Care Unit; LT: Laundry Trolley; MB: Mop Bucket

### Laundry Trolley captures and amplifies the majority of ARGs present in the ICU cages

**Fig 3:**
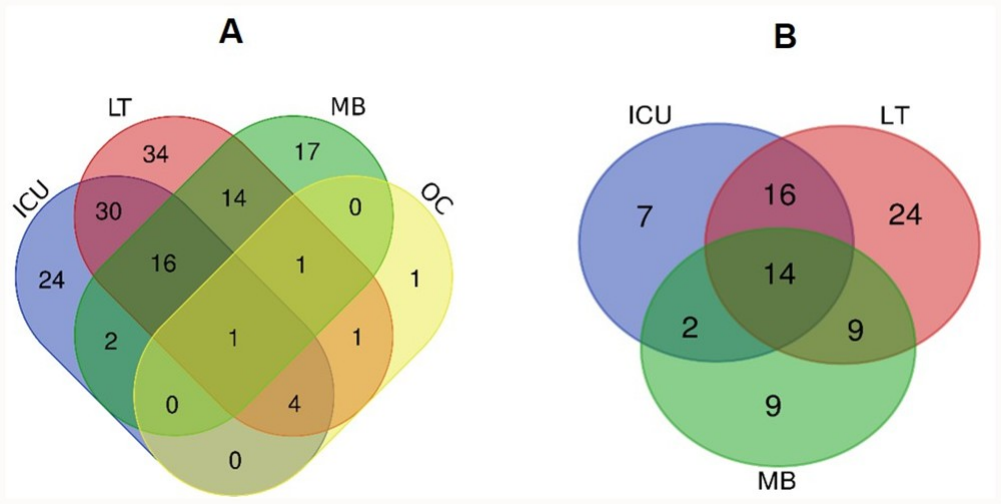
Venn diagrams showing the distribution of shared ARGs in the ICU cages (ICU), Laundry Trolley (LT), Mop Bucket (MB) and the Office Corridor (OC) samples. (A) Total ARG profiles; (B) High-risk ARG profiles (according to the ranking scheme from Martinez et al.).

**Fig 4:**
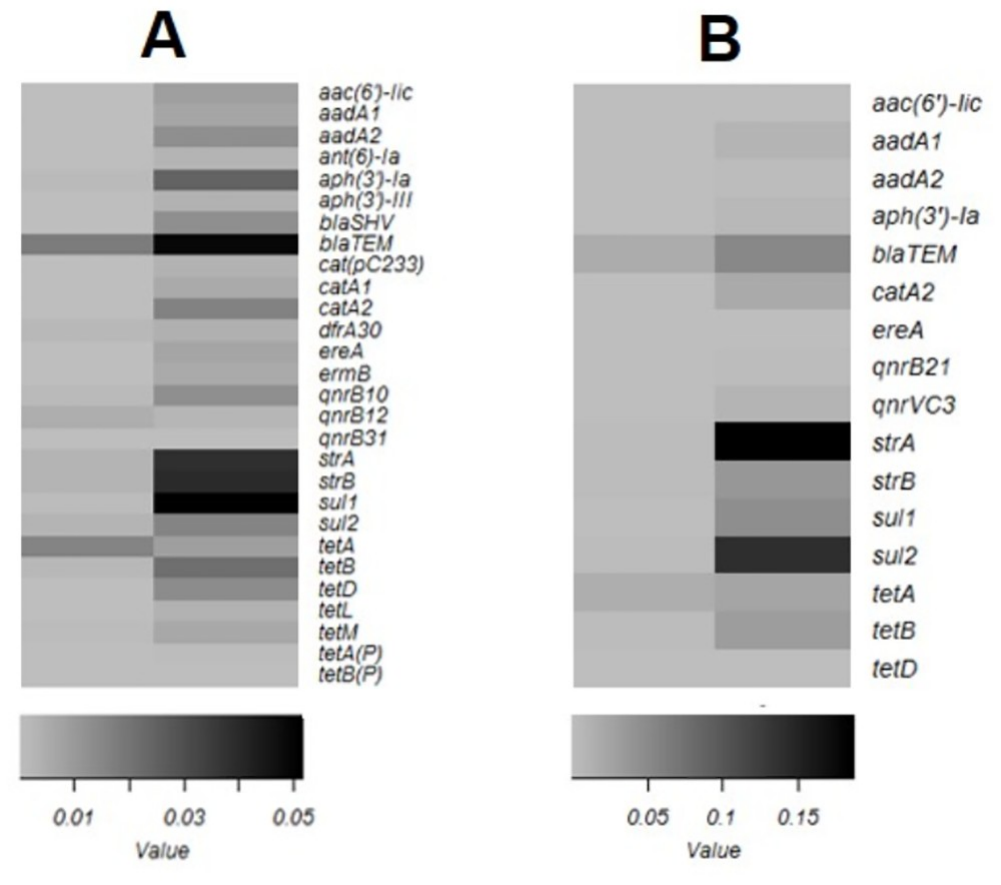
Relative abundances of high-risk genes shared between hospital sites. Comparative abundance between (A) ICU cages and Laundry Trolley (B) ICU cages and Mop Bucket. Grey scale indicates ARG counts per mega base pairs.

Saturation trends of the OTU rarefaction curves indicated that all datasets had sufficient sequencing depth to capture the diversity of the microbial population present in each sample (S1 Fig), allowing the comparative analysis of ARG compositions between samples. The ICU cages, LT, MB and OC samples were estimated to contain 77, 101, 51 and 8 categories of ARGs, respectively. The ABRicate analysis of both MinION long reads and assembled Illumina contigs resulted in the same profile of major ARG categories (Table 3). This confirms the fact that higher error rates in MinION sequence data did not adversely affect the accurate assignment of ARGs. The ICU cages shared 66% of their ARGs with the LT, but only 25% with the MB and 6% with the OC (Fig 3A). Furthermore, the ICU cages shared 77% of high-risk ARGs, as defined by Martinez et al. [56], with the LT, 41% with the MB, and none with the OC sample (Fig 3B). The high-risk ARGs shared between the ICU and other sites included aminoglycoside, sulphonamide, trimethoprim, macrolide, chloramphenicol, and tetracycline resistance genes, as well as ESBLs. The relative abundance of ARGs was highest in the MB sample, with 0.89 copies per total sequence mega base, followed by LT (0.54), ICU cages (0.22) and OC (0.10). High-risk ARGs followed a similar pattern, with 0.83, 0.45 and 0.09 copies per total sequence mega base in the MB, LT and ICU cages samples, respectively. Moreover, higher relative abundances in the LT and MB samples compared to the ICU cages were observed for almost every category of ARG (Fig 4).

**Fig 5:**
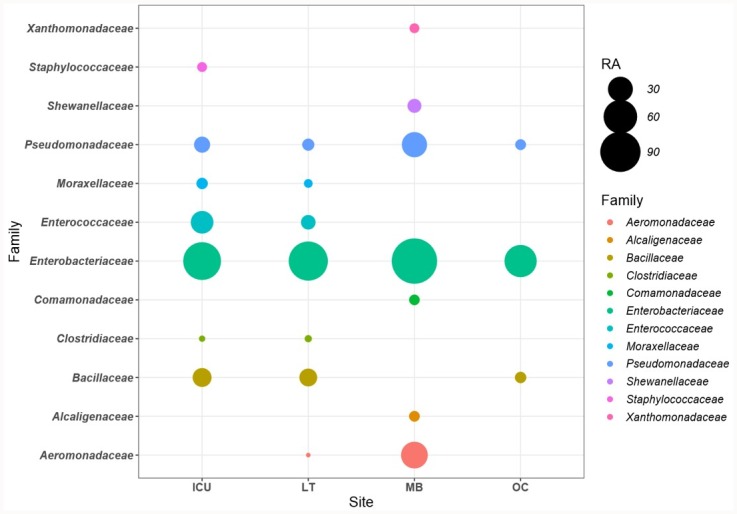
Microbial composition at family level in ICU cages (ICU), Laundry Trolley (LT), Mop Bucket (MB) and Office Corridor (OC) samples. Only the families with relative abundance of >1 copy per mega base pair are represented.

It is difficult to ascertain the impact of the culture enrichment step on the initial population collected, so the conclusions derived from our comparative analysis of relative abundances of ARGs between samples must remain preliminary. However, it is interesting to note that bacterial compositions were similar between the ICU cages and the LT samples, whereas a different taxonomic diversity was observed in MB (Fig 5). These trends could be caused by recurrent contamination events followed by enrichment of resistant bacteria in favourable environmental conditions, and/or by the maintenance of different established microbial populations across the veterinary hospital. Therefore, a possible interpretation of our results is that high-risk ARGs, present at low levels in the immediate environment of the patients (cages), are amplified with their bacterial hosts in the LT, while selection pressures in the MB might create and maintain quite different populations and ARG profiles. To explore these questions, it would be useful to develop reliable culture-free DNA extraction protocols, with sufficient yields to undergo nanopore sequencing, directly from the environmental samples. Some low input DNA library kits and alternative sequence amplification strategies [66] offer promising solutions to address this problem and will be implemented in future experiments.

The patients kept in ICU often require antimicrobial treatments, and the residues of these drugs are likely to accumulate in the cage environment via urine, faeces and other biological materials from the patient. This would add to the selective pressure already applied by the therapeutic course on the flora of the animal, and further increase the risk of developing antimicrobial resistance. The problem could become particularly serious if the laundry trolleys and the mop buckets are less frequently cleaned and disinfected compared to the ICU cages. Veterinary hospital waste represents a major source for re-introduction of resistance and should be monitored regularly. As a practical outcome of this study, the infection control procedures of the veterinary teaching hospital were revised, to enforce the systematic rinsing and drying of buckets and regular disinfection of mop heads and laundry trolleys. Long read sequencing is currently applied to other sections and sites of the veterinary hospital, such as drains, sinks and consultation tables in order to further identify critical control points and better inform future infection control plans.

### Multiple drug resistances are revealed in long reads from veterinary hospital environments

**Fig 6:**
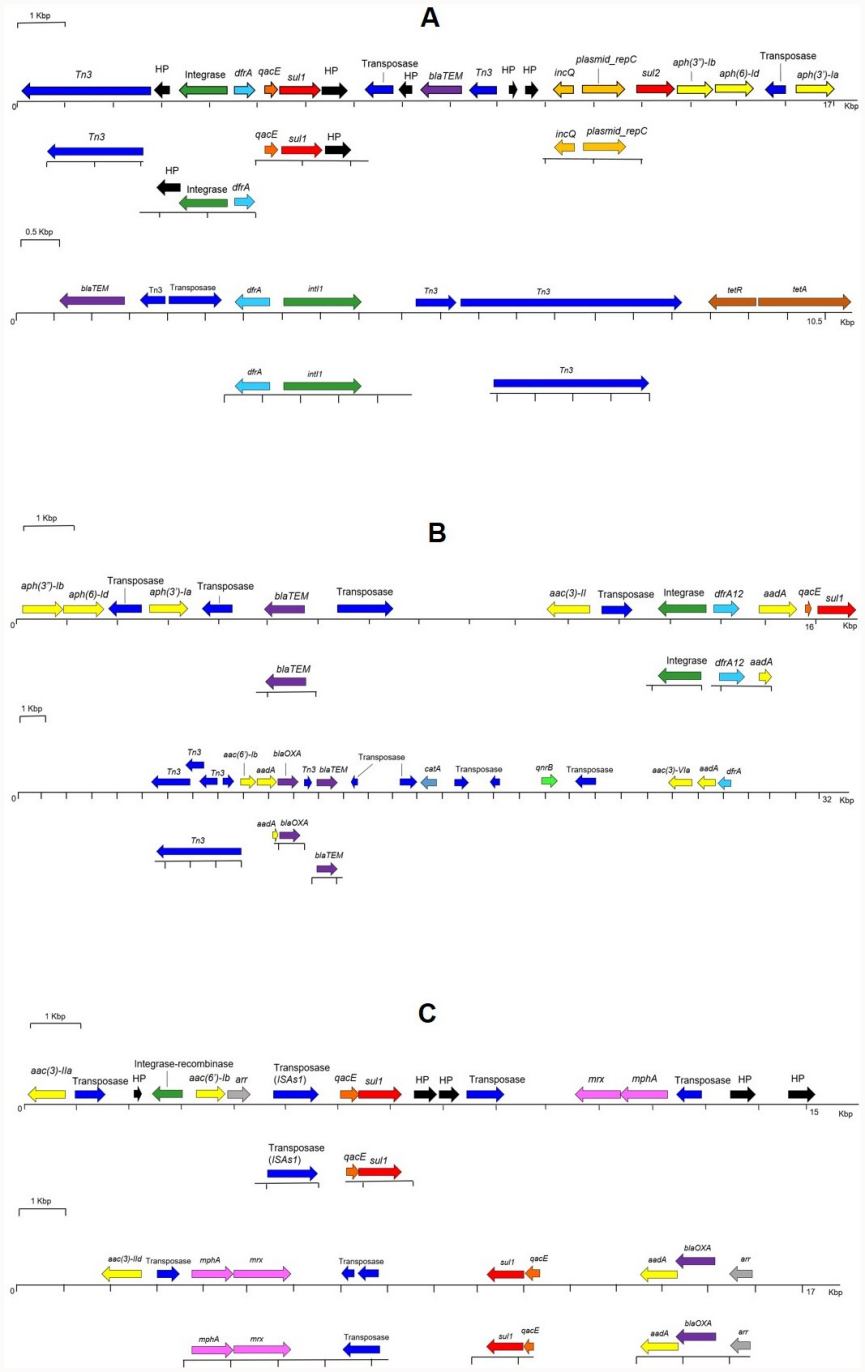
Co-occurrence of ARGs and MGEs in nanopore reads (top) and illumina contigs (bottom). Gene organisation of representative sequences from (A) ICU cages (B) Laundry Trolley and (C) Mop Bucket. Resistance genes, light blue: trimethoprim; red: sulphonamides; purple: beta-lactams; yellow: aminoglycosides; grey: rifamycin; pink: macrolides; blue: chloramphenicol; brown: tetracycline; luminous green: fluoroquinolone; dark orange: quaternary ammonium compounds. Mobile Genetic Elements, dark blue: transposases; green: integrases; light orange: plasmid-associated. HP: Hypothetical Proteins.

Significant proportions of ARG-containing reads carried multiple antibiotic resistances in the LT and MB samples, and to a lesser extent in the ICU cages (Table 5). The majority of these reads also carried sequences related to MGEs. For instance, in LT and MB an *aac(3)-II* gene was located close to an *IS6* transposase on the same read. In ICU cages and LT, *blaTEM* and *aph(3’)-Ia* genes were present adjacent to the *IS6* or *Tn3* family transposase genes, and a *sul1*, *qacE* and *dfrA* gene cluster was found close to an integrase gene, suggesting the presence of an integron. In MB, *qacE* and *sul1* genes were found adjoining *ISAs1* transposase. The *ISAs1* family transposases were previously described for disrupting a gene cassette in class I integron [67] and *sul1/qacE* are well known components of class I integrons [68]. The mapping of the assembled and annotated Illumina contigs to the annotated MinION long reads further confirmed the arrangement of these genes. The length of Illumina contigs were limited and therefore less descriptive when compared to the long reads. This makes the MinION long read sequencing, a much favourable approach to monitor the potential spread of multiple drug resistance in the environment. Some examples of such reads are given in Fig 6A, 6B and 6C. The presence of clinically important ARGs adjacent to MGEs is of particular concern as it reflects the potential risk of disseminating multiple drug resistance within a veterinary clinical environment. While the accumulation of ARGs on individual reads was relatively low in the ICU cages sample, their higher proportion in the LT suggests that exchanges and recombination of ARG occurs in waste environment.

**Table 5:** Sequences carrying multiple antimicrobial resistance genes in the veterinary hospital.

Category	Percentage %		
	ICU	LT	MB
SDR	87.16	69.39	78.92
MDR2	6.09	14.70	17.60
MDR3	2.34	8.96	2.58
MDR≥4	2.06	6.10	0.78
**Total MDR**	**10.50**	**29.76**	**20.96**

SDR: single drug resistance; MDR2: multiple drug resistance due to two different ARGs; MDR3: multiple drug resistance due to three different ARGs; MDR≥4: multiple drug resistance due to four or more different ARGs. ICU: Intensive Care Unit; LT: Laundry Trolley; MB: Mop Bucket

## Conclusions

Nanopore sequencing is a convenient and portable method for routine monitoring of environmental risks associated with infectious agents and antimicrobial resistance in veterinary hospitals. Our findings identified possible transfers of ARGs between interconnected environmental sites and identified waste collection points as significant amplifying reservoirs for clinically important ARGs. This work has led to improving biosecurity practices in the investigated premises and demonstrated the usefulness of rapid DNA sequencing to implement evidence-based operational measures for infection control in veterinary facilities.

## Supporting information

S1 Table Bacterial read counts carrying ARGs in the ICU cages (ICU), Laundry Trolley (LT), Mop Bucket (MB) and Office Corridor (OC). (DOC)

S2 Table Detailed information on independent MinION sequencing runs. (DOCX)

S3 Table Sequence read statistics after filtering for the quality. (DOCX)

S1 Fig Rarefaction curves for number of species detected in samples ICU cages, LT, MB and OC. (DOC)

S1 File Ct values obtained with WaferGen SmartChip Real-time qPCR array. (XLSX)
