# Supplementary material for: Exploration of antibiotic resistance risks in a veterinary teaching hospital with Oxford Nanopore long read sequencing
Source: PLoS One. 2019 May 30;14(5):e0217600. doi: 10.1371/journal.pone.0217600 (PMC6542553; doi:10.1371/journal.pone.0217600)
Supplement: S1 Table — (DOC) [file pone.0217600.s001.doc]

Bacterial read counts carrying ARGs in the ICU cages (ICU), Laundry trolley (LT), Mop bucket (MB) and Office corridor (OC)

| **Taxon^a^** | **Site** | **Resistance genes^b^** | | | | | | | | | |
| --- | --- | --- | --- | --- | --- | --- | --- | --- | --- | --- | --- |
|  |  | **BL** | **MLS** | **GP** | **Tet** | **AG** | **FQ** | **TS** | **Phe** | **Fos** | **Rif** |
| *Enterococcus* | ICU | - | 200 | 146 | 34 | 5 | - | - | 1 | - | - |
|  | LT | - | 67 | 7 | 17 | 14 | - | 1 | 23 | - | - |
|  | MB | - | - | - | - | 1 | - | - | - | - | - |
|  | OC | - | 1 | - | - | - | - | - | - | - | - |
| *Pseudomonas* | ICU | 55 | - | - | 7 | 56 | - | 1 | 20 | 23 | - |
|  | LT | 2 | - | - | 4 | 9 | - | 7 | - | - | - |
|  | MB | 2 | - | - | 5 | 17 | - | 1 | - | - | - |
| *Enterobacteriaceae* | ICU | 170 | 1 | - | 68 | 43 | 44 | 7 | 41 | 13 | - |
|  | LT | 378 | 32 | - | 132 | 321 | 128 | 188 | 74 | 49 | - |
|  | MB | 88 | 22 | - | 71 | 193 | 17 | 99 | 63 | 3 | 20 |
|  | OC | 20 | - | - | - | - | 32 | - | - | 27 | - |
| *Staphylococcus* | ICU | 30 | - | - | 1 | - | 8 | - | - | - | - |
|  | LT | 1 | 1 | - | - | 2 | - | - | 1 | - | - |
| *Acinetobacter* | ICU | 10 | - | - | - | 1 | - | 1 | - | - | - |
|  | LT | 25 | 4 | - | 7 | 42 | - | 17 | 6 | - | - |
|  | MB | 15 | - | - | 4 | 15 | - | 6 | 16 | - | 10 |
| *Xanthobacter* | ICU | - | 1 | - | - | 2 | - | - | - | - | - |
|  | LT | - | 11 | - | - | 15 | - | 8 | - | - | - |
| *Streptococcus* | ICU | - | - | - | 1 | - | - | - | - | - | - |
|  | LT | - | 9 | - | 5 | 19 | - | - | - | - | - |
| *Bacillus* | ICU | - | - | 4 | - | - | - | - | - | - | - |
|  | LT | - | - | 20 | - | - | - | - | - | - | - |
| *Aeromonas* | LT | 11 | - | - | 7 | - | - | - | - | - | - |
|  | MB | 56 | 2 | - | 1 | 9 | - | 3 | 3 | - | - |
| *Shewanella* | ICU | 1 | - | - | - | - | - | - | - | - | - |
|  | LT | 2 | - | - | - | 3 | - | - | - | - | - |
|  | MB | 14 | 1 | - | 1 | 9 | 15 | 11 | 3 | - | 4 |
| *Clostridium* | ICU | - | - | - | 3 | - | - | - | - | - | - |
|  | LT | - | 2 | - | 6 | - | - | - | - | - | - |
| *Vibrio* | ICU | - | - | - | - | - | 2 | - | - | - | - |
|  | LT | - | - | - | - | 2 | - | 2 | - | - | - |
|  | MB | - | - | - | - | 2 | 5 | 2 | - | - | - |
| *Rhizobium* | ICU | - | - | - | - | 1 | - | - | - | - | - |
| *Carnobacterium* | LT | - | - | - | - | - | - | - | 2 | - | - |
| *Lactobacillus* | LT | - | - | - | - | - | - | - | 2 | - | - |
| *Peptoclostridium* | LT | - | - | - | 2 | - | - | - | - | - | - |
| *Zymomonas* | LT | 1 | - | - | - | - | - | - | - | - | - |
| *Pseudoxanthomonas* | LT | - | - | - | - | 1 | - | - | - | - | - |
| *Achromobacter* | MB | - | - | - | - | 2 | - | - | - | - | - |
| *Stenotrophomonas* | MB | 1 | - | - | - | - | - | - | - | - | - |
| *Verminephrobacter* | MB | - | - | - | - | - | - | 1 | - | - | - |

^a^ Taxonomic classification determined by Kraken.

^b^ Antimicrobial: BL: Beta-lactam, MLS: Macrolide/Lincosamide/Streptogramin, GP: Glycopeptide, Tet: Tetracycline, AG: Aminoglycoside, , FQ: Fluroquinolone/Quinolone, TS: Trimethoprim/Sulphonamide, Phe: Phenicol, Fos: Fosfomycin, Rif: Rifamycin.
