# Supplementary material for: Exploration of antibiotic resistance risks in a veterinary teaching hospital with Oxford Nanopore long read sequencing
Source: PLoS One. 2019 May 30;14(5):e0217600. doi: 10.1371/journal.pone.0217600 (PMC6542553; doi:10.1371/journal.pone.0217600)
Supplement: S2 Table — (DOCX) [file pone.0217600.s002.docx]

| **Dataset** | **Sample** | **Date of collection** | **Number of swabs** | **DNA pools** | **Barcodes** | **MinION sequencing run** |
| --- | --- | --- | --- | --- | --- | --- |
| ICU1^a^ | IC1 | 14.11.2016 | 5 | P1 | NA | 1  (22.03.2017) |
|  | IC2 | 28.11.2016 | 5 |  |  |  |
|  | IC3 | 12.12.2016 | 5 |  |  |  |
|  | IC4 | 13.02.2017 | 4 |  |  |  |
| ICU2^a^ | IC1 | 14.11.2016 | 5 | P1 | NA | 2  (01.08.2017) |
|  | IC2 | 28.11.2016 | 5 |  |  |  |
|  | IC3 | 12.12.2016 | 5 |  |  |  |
|  | IC4 | 13.02.2017 | 4 |  |  |  |
| ICU3^a^ | IC1 | 14.11.2016 | 5 | P1 | BC1 | 3  (25.08.2017) |
|  | IC2 | 28.11.2016 | 5 |  |  |  |
|  | IC3 | 12.12.2016 | 5 | P2 | BC2 |  |
|  | IC4 | 13.02.2017  27.02.2017 | 4 |  |  |  |
|  | IC5 |  | 4 | P3 | BC3 |  |
|  | IC6 | 27.03.2017 | 3 |  |  |  |
|  | IC7 | 01.05.2017 | 2 | P4 | BC4 |  |
|  | IC8 | 15.05.2017 | 4 |  |  |  |
| LT1^b^ | L1 | 14.11.2016 | 1 | P1 | NA | 4  (09.05.2017) |
|  | L2 | 28.11.2016 | 1 |  |  |  |
|  | L3 | 12.12.2016 | 1 |  |  |  |
|  | L4 | 13.02.2017 | 1 |  |  |  |
| LT2^b^ | L1 | 14.11.2016 | 1 | P1 | BC1 | 5  (24.01.2018) |
|  | L2 | 28.11.2016 | 1 |  |  |  |
|  | L3 | 12.12.2016 | 1 | P2 | BC2 |  |
|  | L4 | 13.02.2017  27.02.2017 | 1 |  |  |  |
|  | L5 |  | 1 | P3 | BC3 |  |
|  | L6 | 27.03.2017 | 1 |  |  |  |
|  | L7 | 01.05.2017 | 1 | P4 | BC4 |  |
|  | L8 | 15.05.2017 | 1 |  |  |  |
| MB^c^ | M1 | 12.12.2016 | NA | P1 | BC1 | 6  (13.12.2017) |
|  | M2 | 13.02.2017 | NA | P2 | BC2 |  |
|  | M3 | 27.02.2017 | NA |  |  |  |
|  | M4 | 17.03.2017 | NA | P3 | BC3 |  |
|  | M5 | 20.03.2017 | NA |  |  |  |
|  | M6 | 21.03.2017 | NA |  |  |  |
|  | M7 | 23.03.2017 | NA |  |  |  |
|  | M8 | 27.03.2017 | NA |  |  |  |
|  | M9 | 28.03.2017 | NA | P4 | BC4 |  |
|  | M10 | 04.04.2017 | NA |  |  |  |
|  | M11 | 12.04.2017 | NA |  |  |  |
| OC^d^ | OC1 | 28.05.2017 | 1 | P1 | NA | 7  (05.06.2017) |

Detailed information on independent MinION sequencing runs

^a^ The sequence reads of these runs were combined to form the final dataset for ICU cages

^b^ The sequence reads of these runs were combined to form the final dataset for laundry trolley

^c^ The dataset for mop bucket ^d^ The dataset for office corridor NA= not applicable
