# Supplementary material for: Exploration of antibiotic resistance risks in a veterinary teaching hospital with Oxford Nanopore long read sequencing
Source: PLoS One. 2019 May 30;14(5):e0217600. doi: 10.1371/journal.pone.0217600 (PMC6542553; doi:10.1371/journal.pone.0217600)
Supplement: S3 Table — (DOCX) [file pone.0217600.s003.docx]

| **Dataset** | **Size (Gbp)** | **Mean length (Kbp)** | **N50 (Kbp)** |
| --- | --- | --- | --- |
| ICU cages | 6 | 5 | 10 |
| LT | 5 | 6 | 10 |
| MB | 2 | 2 | 4 |
| OC | 1 | 7 | 11 |

Sequence read statistics after filtering for the quality^a^

^a^Reads shorter than 250 bps and reads having less than 8 phred quality score were filtered out
