# Supplementary figures and images for: Exploration of antibiotic resistance risks in a veterinary teaching hospital with Oxford Nanopore long read sequencing
Source: PLoS One. 2019 May 30;14(5):e0217600. doi: 10.1371/journal.pone.0217600 (PMC6542553; doi:10.1371/journal.pone.0217600)

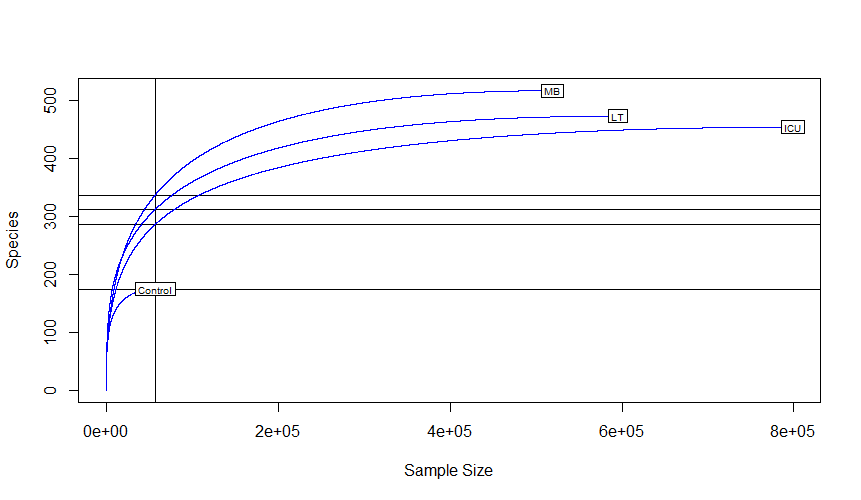


Rarefaction curves for number of species detected in samples ICU cages, LT, MB and OC

Supplement: S1 Fig — (DOC) [file pone.0217600.s004.doc]
